# Exosome-mediated horizontal gene transfer occurs in double-strand break repair during genome editing

**DOI:** 10.1038/s42003-019-0300-2

**Published:** 2019-02-08

**Authors:** Ryuichi Ono, Yukuto Yasuhiko, Ken-ichi Aisaki, Satoshi Kitajima, Jun Kanno, Yoko Hirabayashi

**Affiliations:** 1 Division of Cellular and Molecular Toxicology, Center for Biological Safety and Research (CBSR), National Institute of Health Sciences (NIHS), 3-25-26 Tonomachi, Kawasaki-ku, Kawasaki-shi, Kanagawa-ken 210-9501 Japan; 2 Japan Bioassay Research Center, Japan Organization of Occupational Health and Safety, 2445, Hirasawa, Hadano-shi, Kanagawa-ken 257-0015 Japan

## Abstract

The CRISPR-Cas9 system has been successfully applied in many organisms as a powerful genome-editing tool. Undoubtedly, it will soon be applied to human genome editing, including gene therapy. We have previously reported that unintentional DNA sequences derived from retrotransposons, genomic DNA, mRNA and vectors are captured at double-strand breaks (DSBs) sites when DSBs are introduced by the CRISPR-Cas9 system. Therefore, it is possible that unintentional insertions associated with DSB repair represent a potential risk for human genome editing gene therapies. To address this possibility, comprehensive sequencing of DSB sites was performed. Here, we report that exosome-mediated horizontal gene transfer occurs in DSB repair during genome editing. Exosomes are present in all fluids from living animals, including seawater and breathing mammals, suggesting that exosome-mediated horizontal gene transfer is the driving force behind mammalian genome evolution. The findings of this study highlight an emerging new risk for this leading-edge technology.

## Abstract (web summary)

Ryuichi Ono et al. show that a large number of unintentional trans-species gene transfers can occur when double-strand breaks are introduced by CRISPR-Cas9 in mouse eggs and cell lines. They propose a mechanism of trans-species horizontal gene transfer via double-strand break repair.

## Introduction

Since 2000, three types of genome editing technologies have been developed: zinc-finger nucleases (ZFNs), transcription activator-like effector nucleases (TALENs), and CRISPR-Cas9^1^. Of these, CRISPR-Cas9 features not only the easiest construct design but also high double-strand break (DSB) efficiency; however, CRISPR-Cas9 can cause DSBs at unintended sites^1,2^.

In mouse zygotes, most DSBs introduced by CRISPR-Cas9 are repaired by nonhomologous end joining (NHEJ) without homologous DNA oligos for homologous recombination (HR)^3^. NHEJ-mediated repair of DSBs is prone to error, causing small indels^3^. In 2015, we reported that DSBs introduced by CRISPR-Cas9 can be repaired by the capture of retrotransposon sequences, reverse-transcribed spliced mRNA sequences (RMDR: RT-product-mediated DSB repair) and CRISPR-Cas9 vector sequences (non-RMDR: non-RT-product-mediated DSB repair) in mouse zygotes^4^. Most captured DNA sequences are truncated at their 5′ and 3′ ends. Short microhomologies (1–4 bp) between the captured DNA sequence and the DSB-introduced site were observed in only half of the cases, suggesting that both RMDR and non-RMDR proceed via NHEJ^4^. RMDR and non-RMDR have also been observed in DSBs induced by CRISPR-Cas9 in NIH-3T3 cells^4^.

The capture of DNA sequences was also observed at the DSB site introduced by the I-*Sce*I restriction enzyme in *Saccharomyces cerevisiae*, a human hepatoma cell line and human monocytic leukemia cells and at naturally occurring DSB sites in *Daphnia*, *Drosophila*, and *Aspergillus*^5–11^. Ty1 retrotransposon insertions into DSB sites were induced by I-*Sce*I in HR-deficient *S. cerevisiae*^8,9^. In the case of the hepatoma cell line LMH, I-*Sce*I induced the insertion of truncated infected hepatitis B virus into DSB sites^10^. Endogenous nucleotide sequence insertions were also induced by I-*Sce*I in the human monocytic leukemia cell line U937^11^. In *Daphnia*, *Drosophila*, and *Aspergillus*, greater than half of recent naturally gained introns originated from the repair of staggered DSBs^5–7^.

These capture of unintentional DNA sequences at DSB sites might be an evolutional driving force of mammalian genomes, including horizontal gene transfer. In this report, comprehensive analyses of DSB sites introduced by CRISPR-Cas9 in vivo and in vitro were performed to identify the relationships between DSB repairs and genome evolution and verify the risk for the leading-edge technology. Our results highlight exosome-mediated horizontal gene transfer, which occurs in DSB repair, during genome editing and represents a potential new risk for genome editing.

## Results

### Determination of indels by deep sequencing

**Fig. 1:**
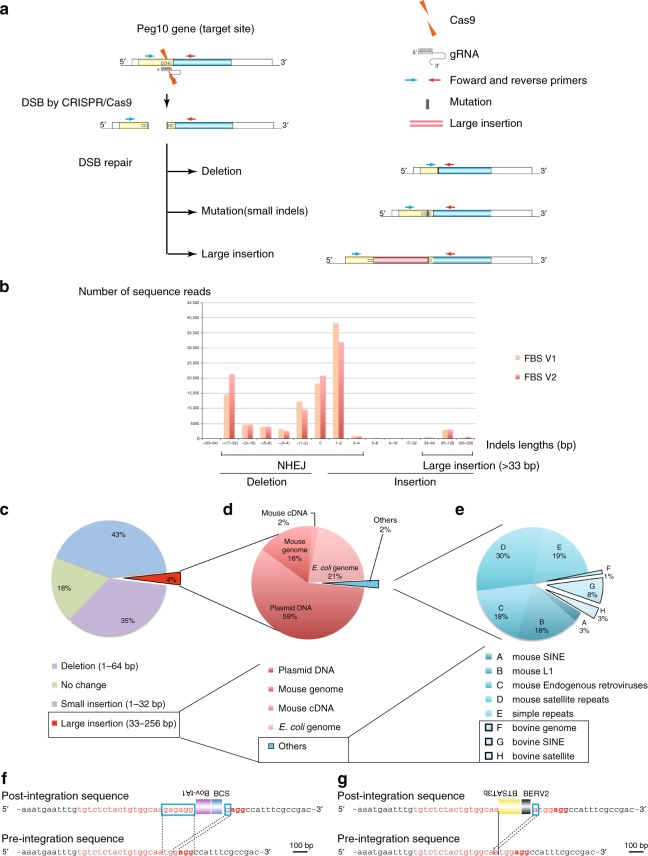
High-throughput indel identification to detect CRISPR-Cas9-induced mutations at the *Peg10* gene locus in NIH-3T3 cells cultured in 10% FBS/DMEM. **a** Schematic representation of the sgRNA, Cas9, and primers. DSBs were repaired with deletions, mutations (small indels), and large insertions. The PCR products amplified using the primers were subjected to high-throughput sequencing. White box: UTR (untranslated region), yellow box: ORF1; blue box: ORF2. **b** The size of the original WT PCR product is presented as 0 bp. The lengths of the insertions are presented as the Plus number, and the lengths of the deletions are presented as the Minus number. Two independent high-throughput sequencing experiments were performed: FBS-V1 and FBS-V2. The total sequence reads of FBS-V2 were normalized to those of FBS-V1. **c** Distribution of indels at CRISPR-Cas9-induced DSB sites in NIH-3T3 cells (FBS-V1). Of the sequence reads, 35% were deletions, and 4% were large insertions (more than 33 bp; red region). **d** Of the large insertions (red region in **c**), 59% corresponded to partial sequences of the transfected plasmid DNA. An additional 16% and 2% of the reads were identical to mouse genomic DNA and mRNA sequences, respectively, and 21% of the large insertions corresponded to *E. coli* genomic DNA. The remaining 2% of the total reads are described in **e** (blue region). **e** 12% of the reads classified as others (blue region in **d**) were from *Bos taurus* (bovine), including genome, SINEs, and satellite DNA sequences. Structures of de novo inserted bovine sequences at the *Peg10* loci (**f**, **g**). Both the post- and preintegration sequences are presented. The sgRNA sequence and the PAM sequences are presented in red and bold red characters, respectively. The black lines indicate the junction sites between pre- and postintegration sequences. The sequences in the blue boxes are overlapping microhomologies and are marked with black dotted lines. Each insertion was truncated at both the 5′ and 3′ ends. **f** Truncated Bov-tA1, BCS, and bovine SINEs were inserted with 6 and 1-bp microhomologies. **g** A truncated BTSAT3b, a bovine satellite, and a partial BERV2, bovine endogenous retrovirus, were inserted with a 1-bp overlapping microhomology

First, we accurately determined the lengths of the indels introduced by the CRISPR-Cas9 system in vivo and in vitro by deep sequencing of PCR products amplified with two primers across the target DSB site (Fig. 1a).

We introduced DSBs at the *Peg10* gene locus by transfecting NIH-3T3 cells with a CRISPR plasmid encoding both Cas9 and gRNA targeting the *Peg10* gene and a PGK-Puro plasmid^4^. After transient selection with puromycin, DNA was extracted from the cells, and PCRs were performed to amplify the region containing the DSB site introduced into *Peg10* (Fig. 1a). Then, the PCR products were subjected to high-throughput next-generation sequencing analyses. Greater than half of the sequence reads contained ±1–2 bp indels as previously described^12^ (Fig. 1b, c, Table 1). These populations may have been repaired by error-prone NHEJ as previously reported^1^. Greater than 90% of the deletions (3–64 bp) exhibited microhomologies (1–4 bp) at the junction, suggesting that these deletions were also mediated by NHEJ^13^ (Fig. 1b, Supplementary Fig. 1, Table 1, Supplementary Data 1).

**Table 1:** Most frequent sequences after CRISPR-Cas9 treatment

Frequent sequences after CRISPR/Cas9 treatment (>2-bp deletions)	Number of sequence reads	Rate of sequences (%)
AGAGACGCCGCAAAATGAATTTGTGTCTCTACTGTG**GC**CGACACGTGTCCAGCGAAAGCCTC	13,263	2.07
AGAGACGCCGCAAAATGAATTTGTGTCTCTACTGTGG**CA**CGTGTCCAGCGAAAGCCTC	11,539	1.80
AGAGACGCCGCAAAATGAATTTGTGTCTCT**AC**ACGTGTCCAGCGAAAGCCTC	11,274	1.76
AGAGACGCCGCAAAATGAATTTGTGTCTCTACTGT**GGC**CATTTCGCCGACACGTGTCCAGCGAAAGCCTC	7264	1.13
AGAGACGCCGCAAAATGAATTTGTGTCTCTACTG**TGG**AGGCCATTTCGCCGACACGTGTCCAGCGAAAGCCTC	6252	0.98
AGAGACGCCGCAAAATGAATTTGTGTCTCTACTGTG**G**ACACGTGTCCAGCGAAAGCCTC	5405	0.84
AGAGACGCCGCAAAATGAATTTGTGTCTCTA**C**GTGTCCAGCGAAAGCCTC	4935	0.77
AGAGACGCCGCAAAATGAATTTGTGTCTCTACTGTGTGGAGGCCATTTCGCCGACACGTGTCCAGCGAAAGCCTC	3279	0.51
AGAGACGCCGCAAAATGAATTTGTGTCTCTACTGTGGCA**A**GGCCATTTCGCCGACACGTGTCCAGCGAAAGCCTC	3116	0.49
AGAGACGCCGCAAAATGAATTTGTGTCTCTACTGTGGCAAGCCATTTCGCCGACACGTGTCCAGCGAAAGCCTC	2251	0.35
AGAGACGCCGCAAAATGAATTTGTGTCTCTACTGTGG**CA**TTTCGCCGACACGTGTCCAGCGAAAGCCTC	2153	0.34
AGAGACGCCGCAAAATGAATTTGTGTCTCTACTGTGGCAACCATTTCGCCGACACGTGTCCAGCGAAAGCCTC	1884	0.29
AGAGACGCCGCAAAATGAATTTGTGTCTCTACTGTGGCA**A**CACGTGTCCAGCGAAAGCCTC	1602	0.25
AGAGACGCCGCAAAATGAATTTGTGTCTCTAC**TGGAG**GCCATTTCGCCGACACGTGTCCAGCGAAAGCCTC	1407	0.22
AGAGACGCCGCAAAATGAATTTGTGTCTCTACTGTGGC**A**CACGTGTCCAGCGAAAGCCTC	1301	0.20
AGAGACGCCGCAAAATGAATTTGTGTCTCTACTGTGGCA**AT**TTCGCCGACACGTGTCCAGCGAAAGCCTC	1271	0.20
AGAGACGCCGCAAAATGAATTTGTGTCTCTACTGT**G**ACACGTGTCCAGCGAAAGCCTC	1180	0.18
AGAGACGCCGCAAAATGAATTTGTGTCTCTACT**GAGGC**CATTTCGCCGACACGTGTCCAGCGAAAGCCTC	1155	0.18
AGAGACGCCGCAAAATGAATTTGTGTCTCTACTGT**GCC**GACACGTGTCCAGCGAAAGCCTC	1143	0.18
AGAGACGCCGCAAAATGAATTTGTGTCTCTACTGTGGCAAATTTCGCCGACACGTGTCCAGCGAAAGCCTC	1072	0.17
AGAGACGCCGCAAAATGAATTTGTGTCTCTACTGTGGCAACGCCGACACGTGTCCAGCGAAAGCCTC	956	0.15
AGAGACGCCGCAAAATGAATTTGTGTCTCTACTGTGGCGGCCATTTCGCCGACACGTGTCCAGCGAAAGCCTC	940	0.15
AGAGACGCCGCAAAATGAATTTGTGTCTCTACTGTTGGAGGCCATTTCGCCGACACGTGTCCAGCGAAAGCCTC	819	0.13
AGAGACGCCGCAAAATGAATTTGTGTCTCTAT**GGAGGC**CATTTCGCCGACACGTGTCCAGCGAAAGCCTC	799	0.12
AGAGACGCCGCAAAATGAATTTGTGTCTCTACTGTGGC**A**GGCCATTTCGCCGACACGTGTCCAGCGAAAGCCTC	777	0.12
AGAGACGCCGCAAAATGAATTTGTGTCTCTACTGTGGCAACATTTCGCCGACACGTGTCCAGCGAAAGCCTC	725	0.11

Indels introduced by CRISPR-Cas9 in NIH-3T3 cells with 10% FBS/DMEM culture medium at *Peg10* gene loci were determined by deep sequencing of PCR products amplified with two primers across the target DSB site. Most frequent sequences are presented. Control sequence (WT) is 5′-AGAGACGCCGCAAAATGAATTTGTGTCTCTACTGTGGCAATGGAGGCCATTTCGCCGACACGTGTCCAGCGAAAGCCTC-3′. Microhomologies that align two broken ends are presented in bold characters

Long insertions (>33 bp) were observed in 4% of sequence reads from DSB-induced NIH-3T3 cells (Fig. 1b, c). Greater than half of the long insertion sequences were derived from plasmid DNA (Fig. 1c, d). In total, 16% and 2% of long insertions were identical to mouse genomic DNA and mRNAs (Fig. 1c, d, Supplementary Data 2). These results are comparable to previous results obtained by gel extraction and subcloning/Sanger sequencing^4^.

### Capture of bovine and *E. coli* genomic DNA by horizontal gene transfer

One of the two novel findings of our high-throughput sequencing analyses is that 21% of the long insertions were derived from *Escherichia coli* genomic DNA. These sequences are identical to the *E. coli* K12 strain, suggesting that they are derived from contamination by the host *E. coli* genomic fragments used to amplify the CRISPR-Cas9 vectors (Fig. 1d). DNA sequences from *E. coli* with or without microhomologies were captured (Supplementary Fig. 2). Finally, 2% of the long insertions mostly (88%) consists of mouse repeats, including mouse short interspersed nuclear elements (SINEs), mouse long interspersed nuclear element-1s (L1s), mouse endogenous retroviruses, and mouse satellite repeats and simple repeats, whereas the remaining 12% of the insertions were derived from bovine genomic DNA, including bovine SINE and bovine satellite repeats (Fig. 1d–g, Table 2a, Supplementary Data 2).

**Table 2:** Number of sequence reads inserted into the *Peg10* DSB locus

Name of inserted DNA	Number of sequence reads	Percentage of total reads with long insertion
**(a)** Number of sequence reads, including murine repetitive elements and bovine DNA sequences, inserted into the *Peg10* DSB locus (FBS V1)		
Mouse SINE	13	0.59%
Mouse L1	83	0.39%
Mouse endogenous retroviruses	84	0.56%
Mouse satellite repeats	137	0.36%
Simple repeats	90	0.36%
Bovine genome	5	0.02%
Bovine SINE	34	0.15%
Bovine satellite repeats	15	0.06%
**(b)** Number of sequence reads, including murine repetitive elements and goat DNA sequences, inserted into the *Peg10* DSB locus		
Mouse SINE	98	0.30%
Mouse L1	83	0.25%
Mouse endogenous retroviruses	103	0.32%
Mouse satellite repeats	319	0.98%
Simple repeats	145	0.44%
Goat genome	10	0.03%
Goat SINE	52	0.16%
Goat satellite repeats	14	0.04%
**(c)** Number of sequence reads, including murine repetitive elements and bovine DNA sequences, inserted into the *Peg10* DSB locus		
Mouse SINE	21	0.19%
Mouse L1	33	0.30%
Mouse endogenous retroviruses	40	0.36%
Mouse satellite repeats	88	0.80%
Simple repeats	47	0.43%
Bovine genome	0	0.00%
Bovine SINE	0	0.00%
Bovine satellite repeats	0	0.00%

### Exosome-mediated horizontal gene transfer

Most of the inserted bovine DNA was derived from bovine satellite DNA sequences, such as BTSAT2, BTSAT3, and BTSAT4, and bovine SINE sequences, such as Bovc-tA2^14^ (Table 2a). Dulbecco’s Modified Eagle Medium (DMEM) containing 10% fetal bovine serum (FBS) was used to culture NIH-3T3 cells. Thus, DNA or RNA from FBS in the form of cell-free DNA/RNA, including exosomal DNA/RNA might be the source of the bovine DNA sequences captured by the DSB sites in the cultured mouse cells^15–21^.

**Fig. 2:**
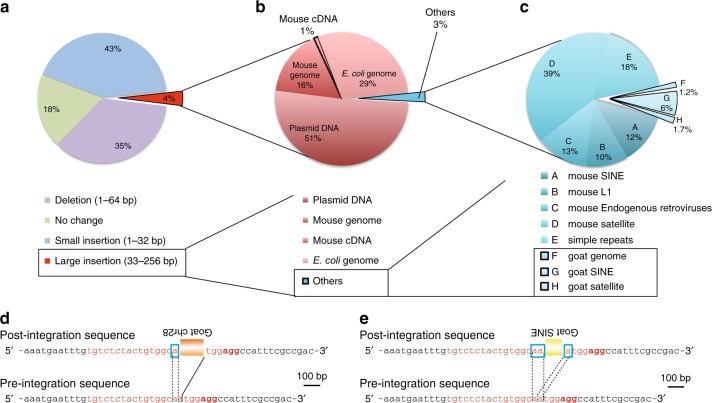
Trans-species horizontal gene transfer at the *Peg10* gene locus from the serum included in the culture medium. **a** Distribution of indels at CRISPR-Cas9-induced DSB sites in NIH-3T3 cells cultured using DMEM containing 10% goat serum instead of FBS. In addition, 38% of the sequence reads were deletions. Large insertions (greater than 33 bp) represented 4% of the total sequence reads (red region). **b** Here, 51% of the large insertions corresponded to partial sequences of the plasmid DNA that was transfected into the NIH-3T3 cells. In addition, 16% and 1% of the reads were identical to mouse genomic DNA and mRNA sequences (MM10), respectively. Moreover, 29% of the large insertions corresponded to *E. coli* genomic DNA. The remaining 3% of the total reads are described in **c** (blue region). **c** Approximately 9% of the reads classified as others were from goat, including the goat genome and goat SINEs and goat satellite DNA. Structures of de novo inserted goat sequences at the *Peg10*-ORF1 loci (**d**, **e**). Both the postintegration site and preintegration sequences (bottom of the panel) are presented. The nucleotide sequences that correspond to the single guide RNA sequence and the PAM sequences are presented in red and bold red characters, respectively. The black lines indicate the junction sites between pre- and postintegration sequences. The sequences in the blue boxes are overlapping microhomologies and are marked with black dotted lines. Each insertion was truncated at both the 5′ and 3′ ends. **d** Partial goat DNA sequences from chromosome 28 were inserted with a 1-bp microhomology. **e** A truncated goat satellite DNA sequence was inserted with a 2-bp overlapping microhomology

**Fig. 3:**
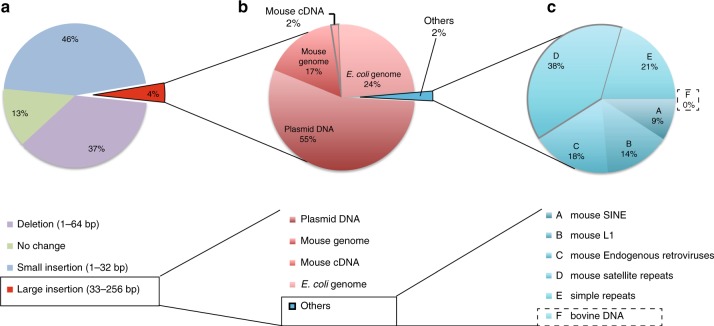
Repression of horizontal gene transfer at the *Peg10* gene locus by using exosome-free FBS. **a** Distribution of indels at CRISPR-Cas9-induced DSB sites in NIH-3T3 cells cultured using DMEM containing 10% exosome-free FBS. Here, 37% of the sequence reads were deletions. Large insertions (greater than 33 bp) represented 4% of the total sequence reads (red region). **b** In addition, 55% of the large insertions corresponded to partial sequences of the plasmid DNA that were transfected into the NIH-3T3 cells. In addition, 17% and 2% of the reads were identical to mouse genomic DNA and mRNA sequences (MM10), respectively. Approximately 24% of the large insertions corresponded to *E. coli* genomic DNA. The remaining 2% of the total reads are described in **c** (blue region). **c** No bovine sequence reads were detected at induced-DSB loci

**Fig. 4:**
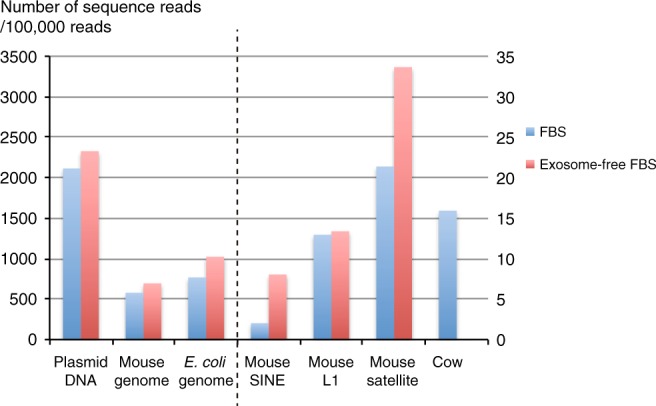
Exosome-mediated trans-species horizontal gene transfer at the *Peg10* gene locus. Comparison of the rate of each type of insertion at induced DSB sites under FBS (FBS V1: blue bars) and exosome-free FBS (red bars) culture conditions

To confirm the possibility of such horizontal gene transfer from the cell culture medium, we repeated these experiments using goat serum instead of FBS (Fig. 2). As noted in the experiments with FBS, mouse genomic DNA, mRNA, or plasmid DNA and *E. coli* genomic DNA were detected as expected (Fig. 2a, b). As expected, goat DNA sequences were captured in the DSB sites of NIH-3T3 cells (Fig. 2c–e, Table 2b, Supplementary Data 3). These data demonstrate that horizontal gene transfer can occur from the serum used in the culture medium. To clarify the origin of the captured bovine DNA sequences, i.e., whether these sequences arose from cell-free nucleic acids or nucleic acids in exosomes, we introduced DSBs by CRISPR-Cas9 in NIH-3T3 cell lines cultured with exosome-free 10% FBS (DMEM), which contains comparable amount of cell-free nucleic acids (Fig. 3, Supplementary Fig. 3). Bovine DNA sequences originating from cell-free nucleic acids should still be introduced at the DSB sites if horizontal gene transfer was mediated by cell-free nucleic acids. In contrast, a reduction in the insertion of bovine DNA sequences in the presence of exosome-free serum would indicate that trans-species gene transfer is mediated by exosomes. The insertion rates of endogenous mouse DNA sequences, vector sequences, and *E. coli* sequences were comparable in cells cultured with exosome-free FBS or normal FBS; however, most of the bovine DNA insertions were abolished by culture with exosome-free 10% FBS/DMEM (Figs. 3a–c and 4, Table 2c, Supplementary Data 4). Furthermore, exosomal RNA collected from FBS by ultracentrifugation and treated with or without RNase and DNase/RNase were comprehensively sequenced. Bovine satellite sequence RNAs and bovine retrotransposon RNAs were highly expressed in FBS under all the conditions, suggesting that bovine satellite sequence RNAs and bovine retrotransposon RNAs were within the exosomes (Supplementary Fig. 4).

**Fig. 5:**
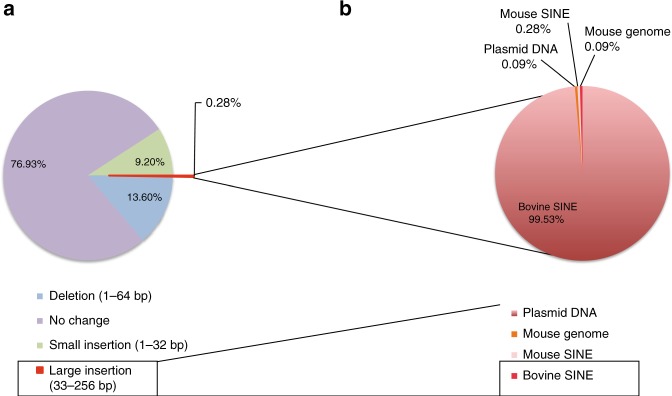
Horizontal gene transfer at the *Peg10* gene locus in mouse zygotes. **a** Distribution of indels at CRISPR/Cas-induced DSB sites in D10 embryo (#20) in which CRISPR-Cas9 mRNA and *Peg10-*ORF1-sgRNA were injected at the zygote stage. Here, 13.6% of the sequence reads were deletions. Large insertions (more than 33 bp) represented 0.26% of the total sequence reads (red region). In addition, 99.53% of large insertions were derived from BTAUL1, a bovine SINE (**b**)

These data support exosome-mediated trans-species gene transfer; however, it is possible that these horizontal gene transfer events were mediated by cell-free nucleic acids. Because exosomes and cell-free nucleic acids are reportedly present in all fluids from living animals, trans-species gene transfer events may also occur in mouse embryos in which DSBs are introduced by injection of CRISPR-Cas9 mRNA into zygotes. Thus, DNA was extracted from day 10 embryos in which CRISPR-Cas9 mRNA and *Peg10* sgRNA were injected at the zygote stage and analyzed by high-throughput sequencing. One of 12 embryos (#20) captured BTAUL1, a bovine SINE (Fig. 5a, b, Supplementary Fig. 5, Supplementary Data 5). The KSOM medium used to culture the mouse zygotes contains bovine serum albumin (BSA) fraction V, which may contain exosomes or cell-free nucleic acids.

## Discussion

In this report, we demonstrated that horizontal gene transfer assisted by CRISPR-Cas9 occurs in NIH-3T3 cells and mouse embryos. This phenomenon might be the driving force behind mammalian genome evolution. In fact, mice with fusions between the murine *Peg10* gene and a bovine SINE were obtained (Supplementary Fig. 5). A number of possible trans-species horizontal gene transfer events have been reported in mammals. Chromodomains (chromatin organization modifier), a protein structural domain, are highly conserved in chromoviruses, and SCAN domains might originate from GYPSYDR-1 retrotransposons. *Sirh*-family genes, which are conserved in mammals, contain a gag-like domain from the Ty3/Gypsy-type retrotransposon of fugu fish^22–26^. Recently, in silico analyses demonstrated horizontal transfer of BovB (non-LTR retrotransposon from *Bos taurus*) and L1 retrotransposons (*B. taurus*) in eukaryotes^27^. In this study, we revealed that BovB and L1 were abundant in exosomes and that goat BovB was horizontally transferred into mouse NIH-3T3 cells. These data support that horizontal gene transfer events are mediated by exosomes.

CRISPR-Cas9 itself exhibits some propensity for inducing off-target mutations^2^. The DSBs produced by CRISPR-Cas9, whether on target or off target, were repaired by the capture of unintentional DNA sequences^2^. Although the risk of unintentional insertions is greater than 4%, considerable efforts have focused on reducing off-target effects. The pair of CRISPR-Cas9 D10A (nickase) and a high-fidelity CRISPR-Cas9 nuclease reduce genome-wide off-target effects^28,29^. These efforts hold promise because DSBs at off-target sites could be neglected. However, unintentional insertions at on-target DSB site cannot be suppressed by these off-target-reducing methods. Therefore, gene therapy using these genome-editing technologies may capture unintentional insertions. DSBs are typically repaired by NHEJ or HR. NHEJ is the predominant pathway in mammals^30,31^ and *Drosophila*^32,33^, whereas HR is the major pathway in *S. cerevisiae*^34^. Another DSB repair mechanism, microhomology-mediated end-joining (MMEJ), repairs DSBs via the use of substantial microhomology. MMEJ uses microhomologies of 5–25 bp during the alignment of two broken ends, whereas NHEJ frequently proceeds through the annealing of short (1–4 bp) microhomologies^13^. Most of the insertion sequences identified in the present study displayed short microhomologies (1–4 bp) or no microhomology with the introduced DSB site, suggesting that they were captured by NHEJ rather than MMEJ.

SCR7, an inhibitor of NHEJ, improves the efficiency of HR in genome editing^35,36^. Increasing the efficiency of HR may be a key strategy to reduce the risk of unintended insertions.

## Methods

### Animals

All animal studies were conducted in accordance with the guidelines approved by the animal care committee of the National Institute of Health Sciences (No. 1004). The animal welfare committee of National Institute of Health Sciences (No. 539) approved the protocol. Animals had access to a standard chow diet and water ad libitum and were housed in a pathogen-free barrier facility with a 12L:12D cycle, as previously described^4^.

### Plasmid preparation

The plasmids expressing both *hCas*9 and *Peg10*-ORF1-sgRNA were prepared as previously described^4^. Briefly, *Peg10*-ORF1-sgRNA (5′-TGTCTCTACTGTGGCAATGG-3′) oligo DNA was ligated into the *Bbs*I site of pX330 (http://www.addgene.org/42230/). Plasmid preparations were performed using the QIAGEN Plasmid Maxi kit (QIAGEN, Hilden, Germany). All oligo sequences in this study are shown in Table 3.

**Table 3:** List of oligo DNA sequences for sgRNA, IVT, and amplicon sequence

sgRNA sequence	
*Peg10*-ORF1-sgRNA3	TGTCTCTACTGTGGCAATGG
PCR primers for IVT	
*Peg10*-ORF1-IVT-F	TGTAATACGACTCACTATAGGGTGTCTCTACTGTGGCAATGG
*Peg10*-ORF1-IVT-R	AAAAGCACCGACTCGGTGCC
PCR primers for amplicon sequence	
*Peg10* F	AATGATACGGCGACCACCGAGATCTACACNNNNNNNNTCGTCGGCAGCGTCAGATGTGTATAAGAGACAGagagacgccgcaaaatgaat
*Peg10* R	CAAGCAGAAGACGGCATACGAGATNNNNNNNNGTCTCGTGGGCTCGGAGATGTGTATAAGAGACAGgaggctttcgctggacac

NNNNNNNN = Illumina barcode N sequence

### Production of hCas9 mRNA and *Peg10*-ORF1-sgRNA

To produce the Cas9 mRNA, the T7 promoter was added to the Cas9 coding region of the pX330 plasmid by PCR amplification, as previously described^3^. Briefly, the T7-Cas9 PCR product was gel purified and used as the template for in vitro transcription (IVT) using the mMESSAGE mMACHINE T7 ULTRA kit (Thermo Fisher Scientific, Waltham, MA). The T7 promoter was added to the *Peg10*-ORF1-sgRNA region of the pX330 plasmid by PCR purification using the following primers as previously described: *Peg10*-ORF1-IVT-F (TGTAATACGACTCACTATAGGGTGTCTCTACTGTGGCAATGG) and IVT-R (AAAAGCACCGACTCGGTGCC)^4^.

The T7-sgRNA PCR product was gel purified and used as the template for IVT using the MEGAshortscript T7 kit (Thermo Fisher Scientific, Waltham, MA). Both the Cas9 mRNA and *Peg10*-ORF1-sgRNA were treated with DNase to eliminate template DNA, purified using the MEGAclear kit (Thermo Fisher Scientific, Waltham, MA), and eluted into RNase-free water as previously described^4^.

### Cell culture

NIH-3T3 cells (RIKEN BRC-Cell Bank: RBRC-RCB2767) were cultured with 10% FBS (Invitrogen)/DMEM, 10% goat serum (Cosmo Bio, Tokyo, Japan)/DMEM or 10% exosome-free FBS (System Biosciences, Palo Alto, CA)/DMEM. pX330-*Peg10*-ORF1 plasmid and pGK-puro plasmid (500 ng each) were introduced into 2 × 10^5^ NIH-3T3 cells/well in a six-well plate using Lipofectamine LTX reagent (Thermo Fisher Scientific, Waltham, MA). At 24 h after transfection, 10 μg/ml puromycin (Thermo Fisher Scientific, Waltham, MA) was added to the wells. Two days after transfection, the cells were collected, and genomic DNA was extracted as previously described^4^.

### Exosome collection and exosome RNA isolation

Exosomes were prepared by a stepwise centrifugation–ultracentrifugation method as described previously with minor modifications^17^. Briefly, 1.4 ml FBS was centrifuged at 10,000×*g* for 30 min to remove the cell debris and then centrifuged at 100,000×*g* for 70 min using a TLA-55 rotor (BECKMAN COULTER, Indianapolis, IN). The pellets were washed twice with phosphate-buffered saline (PBS) and then resuspended in 87.5 μL of PBS as exosome-enriched fractions. These exosome-enriched fractions were treated with RNase and RNase/DNase to remove extraneous nucleic acids outside of exosomes. Then, the exosome fraction with and without RNase and RNase/DNase treatment was mixed with 700 μL of QIAzol Lysis reagent (QIAGEN, Hilden, Germany), and the aqueous phase was collected by adding chloroform. After the addition of ethanol to the aqueous phase, total RNA was purified using RNeasy Mini Elute Spin Columns (QIAGEN, Hilden, Germany). The RNA sample was in 14 μL of nuclease-free water. The concentration of RNA was determined using a NanoDrop spectrophotometer (Thermo Fisher Scientific, Waltham, MA), and the quality of RNA was analyzed using an Agilent 2100 Bioanalyzer and RNA pico chips (Agilent Technologies, Palo Alto, CA).

### PCR and DNA sequencing

For analyses of unintentional sequence insertion associated with DSB repairs, genomic DNA was prepared from the embryonic yolk sac or cultured cells using the DNeasy kit (QIAGEN, Hilden, Germany). The identity of the indels induced by DSB repair was confirmed by PCR and subsequent next-generation sequencing using MiSeq (Illumina Inc., San Diego, CA). The following primers were used: *Peg10* F (5′-AATGATACGGCGACCACCGAGATCTACACNNNNNNNNTCGTCGGCAGCGTCAGATGTGTATAAGAGACAGagagacgccgcaaaatgaat-3′; NNNNNNNN = Illumina barcode S sequence) and *Peg10* R (5′-CAAGCAGAAGACGGCATACGAGATNNNNNNNNGTCTCGTGGGCTCGGAGATGTGTATAAGAGACAGgaggctttcgctggacac-3′; NNNNNNNN = Illumina barcode N sequence) as previously described^4^.

A mixture of 1× *Ex*Taq buffer (Takara Bio, Kusatsu, Japan), 2.5 mM dNTPs, primers and 2.5 U of *Ex*Taq (Takara Bio, Kusatsu, Japan) was subjected to 32 PCR cycles of 96 °C for 15 s, 65 °C for 30 s, and 72 °C for 30 s in a Bio-Rad C1000 Touch system. Each PCR product was purified using an Ampure XP (BECKMAN COULTER, Indianapolis, IN) as previously described^4^.

For analyses of exosomes from FBS, exosome cDNA libraries were synthesized with SMARTer smRNA-Seq Kit for Illumina (Takara Bio, Kusatsu, Japan). The concentration of the PCR products with DSB repair and cDNA synthesized from exosome RNA were quantified using a Kapa Library Quantification kit (Roche, Basel, Switzerland). These products (8 pM) were then subjected to 300 cycles of paired-end index sequencing (total 600 cycles) on an Illumina MiSeq sequencer according to the manufacturer’s instructions (Illumina Inc., San Diego, CA). All the sequence data were converted to FASTQ format by using Illumina BaseSpace (https://basespace.illumina.com/home/index).

### Evaluation of cell-free nucleic acids in FBS and exosome-free FBS

Briefly, 24 ml of FBS and exosome-free FBS were centrifuged at 10,000×*g* for 30 min to remove the cell debris and cell-free nucleic acids were purified via a phenol-chloroform procedure. Then, ethanol precipitation with glycogen was performed and eluted into DNase/RNase-free water. The concentration and quality of cell-free nucleic acids were determined by using a NanoDrop spectrophotometer (Thermo Fisher Scientific, Waltham, MA) and Agilent 2100 Bioanalyzer and DNA HS chips (Agilent Technologies, Palo Alto, CA), respectively.

### One-cell embryo injection

C57BL/6J × CBA F1 female mice (4 weeks) (Charles River Japan, Yokohama, Japan) were superovulated, and IVF was performed using C57BL/6J male mice sperm. Then, 50 ng/μL Cas9 mRNA and 25 ng/μL *Peg10*-ORF1-sgRNA were injected into the cytoplasm of 147 fertilized eggs. The eggs were cultivated overnight in KSOM, and 55 fertilized eggs were then transferred into the oviducts of pseudopregnant MCH females (CLEA Japan, Inc. Tokyo, Japan) as previously described^4^.

### Determination of sequence length distribution

All analyses were performed using Galaxy (https://usegalaxy.org). FASTQ files were filtered by the FILTER By Quality program with default parameters. Paired-end reads were merged using the PEAR program and default parameters, and assembled reads with the *Peg10*-F sequence at the 5′ end and the *Peg10*-R sequence at the 3′ end were filtered by the Barcode Splitter program. The lengths of the filtered sequences were counted by the Compute sequence length program. Frequent reads were identified by FastQC.

### Sequence analyses

PCR products longer (>32 bp) than WT were analyzed by the BLASTN program from the NCBI server (http://www.ncbi.nlm.nih.gov/BLAST/) and the CENSOR program from the GENETIC INFORMATION RESEARCH INSTITUTE (http://www.girinst.org/censor/index.php). The reference sequences are *Mus musculus* (mouse): MM10 (NCBI) for mouse genome, GRCm38 (NCBI) for mouse cDNA and ENSMUST (Ensembl) for mouse mRNA; *B. taurus* (bovine): bosTau7 (NCBI) for bovine genome; *Capra hircus* (goat): CHIR_1.0 (NCBI) for goat genome; and *E. coli* str.K-12 (NCBI) for *E. coli* genome. These data were obtained from the NCBI (https://www.ncbi.nlm.nih.gov/genome/) and Ensembl (https://asia.ensembl.org/info/data/ftp/index.html) databases.

### Reporting summary

Further information on experimental design is available in the Nature Research Reporting Summary linked to this article.

## Supplementary information

Description of Additional Supplementary Files

Reporting Summary

Supplementary Information

Supplementary Data 1

Supplementary Data 2

Supplementary Data 3

Supplementary Data 4

Supplementary Data 5

## Data Availability

The authors declare that the data supporting the findings of this study are available within the article and its Supplementary Information files.

## References

[1] Gaj T, Gersbach CA, Barbas CF (2013). ZFN, TALEN, and CRISPR/Cas-based methods for genome engineering. Trends Biotechnol..

[2] Fu Y (2013). High-frequency off-target mutagenesis induced by CRISPR-Cas nucleases in human cells. Nat. Biotechnol..

[3] Wang H (2013). One-step generation of mice carrying mutations in multiple genes by CRISPR/Cas-mediated genome engineering. Cell.

[4] Ono R (2015). Double strand break repair by capture of retrotransposon sequences and reverse-transcribed spliced mRNA sequences in mouse zygotes. Sci. Rep..

[5] Li W, Tucker AE, Sung W, Thomas WK, Lynch M (2009). Extensive, recent intron gains in Daphnia populations. Science.

[6] Farlow A, Meduri E, Dolezal M, Hua L, Schlotterer C (2010). Nonsense-mediated decay enables intron gain in Drosophila. PLoS Genet..

[7] Zhang LY, Yang YF, Niu DK (2010). Evaluation of models of the mechanisms underlying intron loss and gain in Aspergillus fungi. J. Mol. Evol..

[8] Moore JK, Haber JE (1996). Capture of retrotransposon DNA at the sites of chromosomal double-strand breaks. Nature.

[9] Teng SC, Kim B, Gabriel A (1996). Retrotransposon reverse-transcriptase-mediated repair of chromosomal breaks. Nature.

[10] Bill CA, Summers J (2004). Genomic DNA double-strand breaks are targets for hepadnaviral DNA integration. Proc. Natl. Acad. Sci. U.S.A..

[11] Onozawa M (2014). Repair of DNA double-strand breaks by templated nucleotide sequence insertions derived from distant regions of the genome. Proc. Natl. Acad. Sci. U.S.A..

[12] Yang Z (2015). Fast and sensitive detection of indels induced by precise gene targeting. Nucleic Acids Res..

[13] McVey M, Lee SE (2008). MMEJ repair of double-strand breaks (director’s cut): deleted sequences and alternative endings. Trends Genet..

[14] Kohany O, Gentles AJ, Hankus L, Jurka J (2006). Annotation, submission and screening of repetitive elements in Repbase: RepbaseSubmitter and Censor. BMC Bioinformatics.

[15] Alberry M (2007). Free fetal DNA in maternal plasma in anembryonic pregnancies: confirmation that the origin is the trophoblast. Prenat. Diagn..

[16] Raposo G (1996). B lymphocytes secrete antigen-presenting vesicles. J. Exp. Med..

[17] Valadi H (2007). Exosome-mediated transfer of mRNAs and microRNAs is a novel mechanism of genetic exchange between cells. Nat. Cell Biol..

[18] Lawrie CH (2009). Aberrant expression of microRNA biosynthetic pathway components is a common feature of haematological malignancy. Br. J. Haematol..

[19] de Jong, O. G. et al. Cellular stress conditions are reflected in the protein and RNA content of endothelial cell-derived exosomes. *J. Extracell. Vesicles* **1**, 18396 (2012). 10.3402/jev.v1i0.18396 PMC3760650 24009886

[20] Yoshioka, Y. et al. Comparative marker analysis of extracellular vesicles in different human cancer types. *J. Extracell. Vesicles* **2**, 20424 (2013). 10.3402/jev.v2i0.20424 PMC3760642 24009892

[21] Yanez-Mo M (2015). Biological properties of extracellular vesicles and their physiological functions. J. Extracell. Vesicles.

[22] Ono R (2001). A retrotransposon-derived gene, PEG10, is a novel imprinted gene located on human chromosome 7q21. Genomics.

[23] Ono R (2003). Identification of a large novel imprinted gene cluster on mouse proximal chromosome 6. Genome Res..

[24] Ono R (2006). Deletion of Peg10, an imprinted gene acquired from a retrotransposon, causes early embryonic lethality. Nat. Genet..

[25] Sekita Y (2008). Role of retrotransposon-derived imprinted gene, Rtl1, in the feto-maternal interface of mouse placenta. Nat. Genet..

[26] Naruse M (2014). Sirh7/Ldoc1 knockout mice exhibit placental P4 overproduction and delayed parturition. Development.

[27] Ivancevic AM, Kortschak RD, Bertozzi T, Adelson DL (2018). Horizontal transfer of BovB and L1 retrotransposons in eukaryotes. Genome Biol..

[28] Ran FA (2013). Double nicking by RNA-guided CRISPR Cas9 for enhanced genome editing specificity. Cell.

[29] Kleinstiver BP (2016). High-fidelity CRISPR-Cas9 nucleases with no detectable genome-wide off-target effects. Nature.

[30] Takata M (1998). Homologous recombination and non-homologous end-joining pathways of DNA double-strand break repair have overlapping roles in the maintenance of chromosomal integrity in vertebrate cells. EMBO J..

[31] Rebuzzini P (2005). New mammalian cellular systems to study mutations introduced at the break site by non-homologous end-joining. DNA Repair.

[32] Preston CR, Flores CC, Engels WR (2006). Differential usage of alternative pathways of double-strand break repair in Drosophila. Genetics.

[33] Beumer KJ (2008). Efficient gene targeting in Drosophila by direct embryo injection with zinc-finger nucleases. Proc. Natl. Acad. Sci. U.S.A..

[34] Jeggo PA (1998). DNA breakage and repair. Adv. Genet..

[35] Chu VT (2015). Increasing the efficiency of homology-directed repair for CRISPR-Cas9-induced precise gene editing in mammalian cells. Nat. Biotechnol..

[36] Maruyama T (2015). Increasing the efficiency of precise genome editing with CRISPR-Cas9 by inhibition of nonhomologous end joining. Nat. Biotechnol..

