# Supplementary material for: Exosome-mediated horizontal gene transfer occurs in double-strand break repair during genome editing
Source: Commun Biol. 2019 Feb 8;2:57. doi: 10.1038/s42003-019-0300-2 (PMC6368560; doi:10.1038/s42003-019-0300-2)
Supplement: Supplementary file 1 — Description of Additional Supplementary Files [file 42003_2019_300_MOESM1_ESM.docx]

**Supplementary Data 1** Distribution of indels at CRISPR/Cas-induced DSB sites in NIH-3T3 cells.

The PCR products amplified using the two primers, Peg10 F and Peg10 R, were subjected to high-throughput sequencing. The size of the original WT PCR product is 79bp.

**Supplementary Data 2** Sequence reads with insertions at CRISPR/Cas-induced DSB sites in NIH-3T3 cells cultured in 10% FBS/DMEM (FBS V1).

The WT PCR products amplified using the two primers, Peg10 F and Peg10 R, were "agagacgccgcaaaatgaatttgtgtctctactgtggcaatggaggccatttcgccgacacgtgtccagcgaaagcctc".

**Supplementary Data 3** Sequence reads with insertions at CRISPR/Cas-induced DSB sites in NIH-3T3 cells cultured in 10% goat serum/DMEM.

The WT PCR products amplified using the two primers, Peg10 F and Peg10 R, were "agagacgccgcaaaatgaatttgtgtctctactgtggcaatggaggccatttcgccgacacgtgtccagcgaaagcctc".

**Supplementary Data 4** Sequence reads with insertions at CRISPR/Cas-induced DSB sites in NIH-3T3 cells cultured in 10% exosome-free FBS/DMEM.

The WT PCR products amplified using the two primers, Peg10 F and Peg10 R, were "agagacgccgcaaaatgaatttgtgtctctactgtggcaatggaggccatttcgccgacacgtgtccagcgaaagcctc".

**Supplementary Data 5** Sequence reads with insertions at CRISPR/Cas-induced DSB sites in D10 embryo (#20) in which CRISPR-Cas9 mRNA and Peg10-ORF1-sgRNA were injected at the zygote stage.

The WT PCR products amplified using the two primers, Peg10 F and Peg10 R, were "agagacgccgcaaaatgaatttgtgtctctactgtggcaatggaggccatttcgccgacacgtgtccagcgaaagcctc".
