# Supplementary material for: Exosome-mediated horizontal gene transfer occurs in double-strand break repair during genome editing
Source: Commun Biol. 2019 Feb 8;2:57. doi: 10.1038/s42003-019-0300-2 (PMC6368560; doi:10.1038/s42003-019-0300-2)
Supplement: Supplementary file 3 — Supplementary Information [file 42003_2019_300_MOESM3_ESM.pdf]

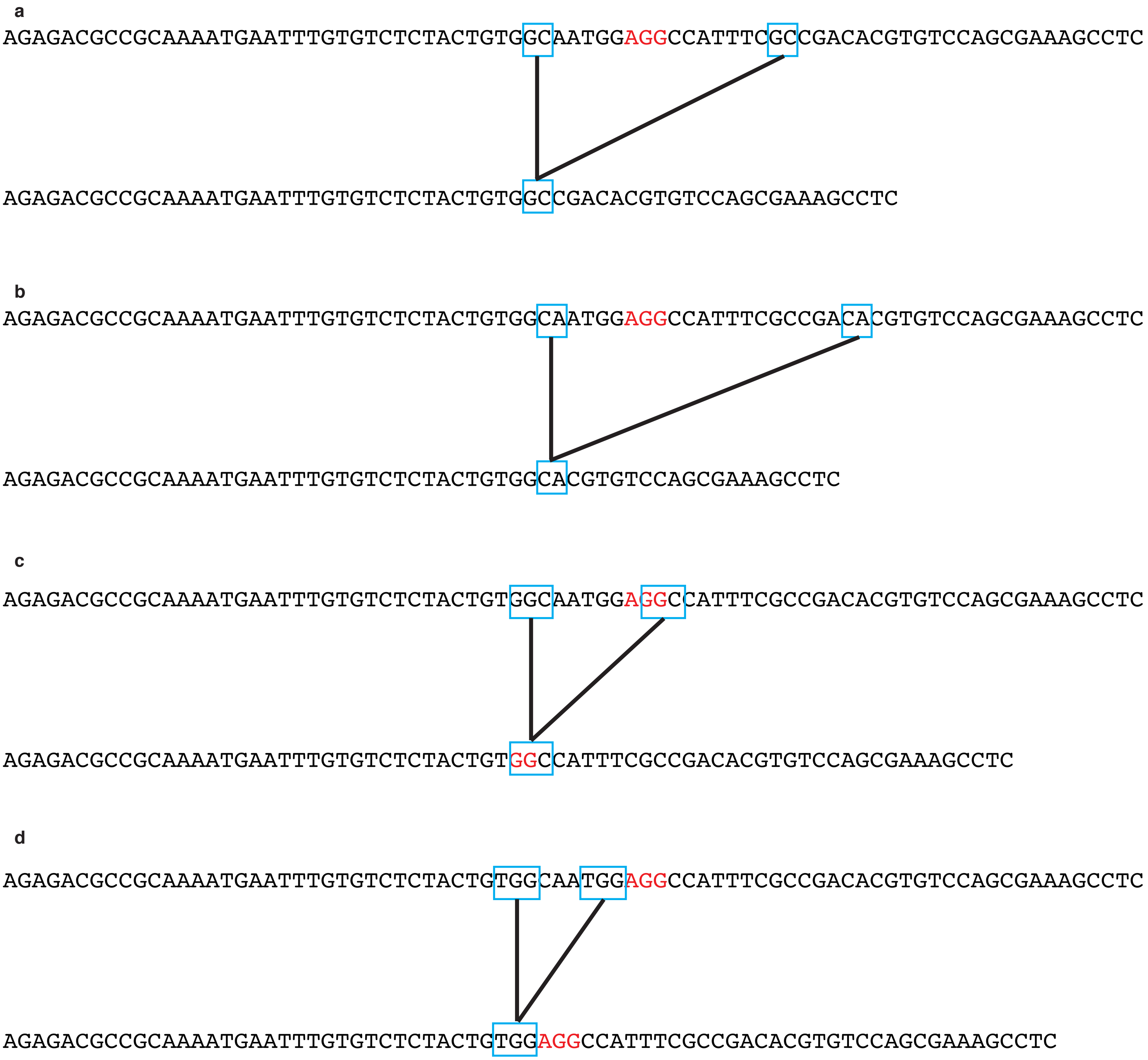

Supplementary Figure 1

The most-frequent reads in CRISPR-Cas9-induced DSB sites at the *Peg10* gene locus in NIH-3T3 cells (>2-bp deletion). (**a**) The most frequent sequence reads (13263 reads, 2.07% of total reads) are presented. Both the WT sequence (upper) and the deleted sequences (bottom) are presented. The PAM sequences are presented in red characters. The nucleotide sequences that correspond to the microhomology are presented in blue boxes. (**b**) The second-most frequent sequence reads (11539 reads, 1.80% of total reads) are presented. Both the WT sequence (upper) and the deleted sequences (bottom) are presented. The PAM sequences are presented in red characters. The nucleotide sequences that correspond to the microhomology are presented in blue boxes. (**c**) The third-most frequent sequence reads (7264 reads, 1.13% of total reads) are presented. Both the WT sequence (upper) and the deleted sequences (bottom) are presented. The PAM sequences are indicated in red characters. The nucleotide sequences that correspond to the microhomology are shown in blue boxes. (**d**) The fourth-most frequent sequence reads (6252 reads, 0.98% of total reads) are presented. Both the WT sequence (upper) and the deleted sequences (bottom) are shown. The PAM sequences are indicated by red characters. The nucleotide sequences that correspond to the microhomology are indicated in blue boxes.

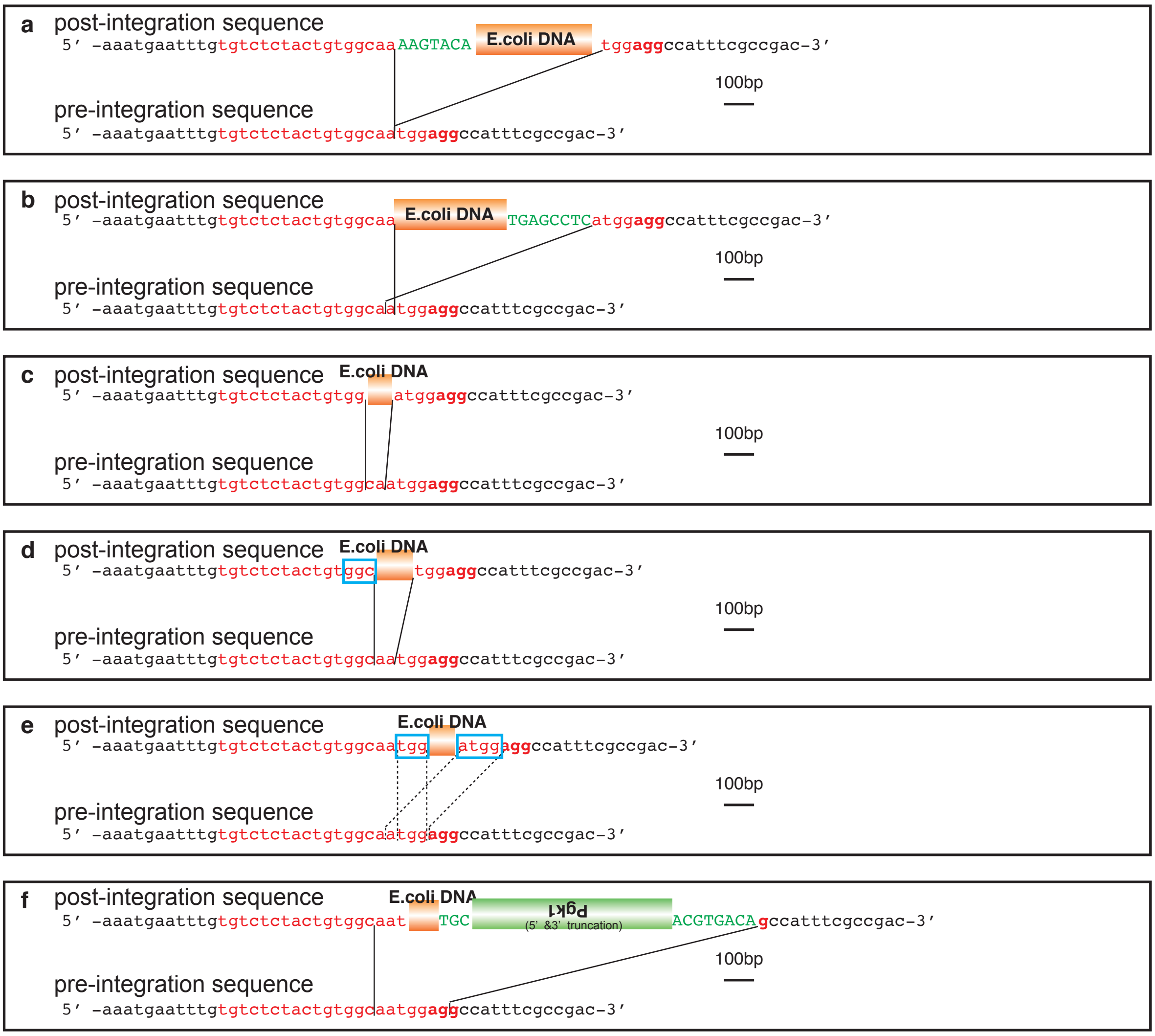

Supplementary Figure 2

Structure of the captured *E. coli* DNA sequences associated with DSB repair.

(**a**-**f**) De novo inserted *E. coli* DNA sequences at the *Peg10*-ORF1 introduced DSB locus in NIH-3T3 cells. Both the postintegration site and preintegration sequences (bottom) are presented. The nucleotide sequences that correspond to the single guide RNA sequence and the PAM sequences are noted in red and bold red characters, respectively. The black lines indicate the junction sites between the pre- and postintegration sequences. The sequences in the blue boxes are overlapping microhomologies and are marked with black dotted lines. Short sequences of unknown origin are presented in green. Each insertion was truncated at both the 5′ and 3′ ends but exhibited distinct features. Both *E. coli* DNA and *Pgk1* mRNA sequences were captured at the same allele. (**f**)

a

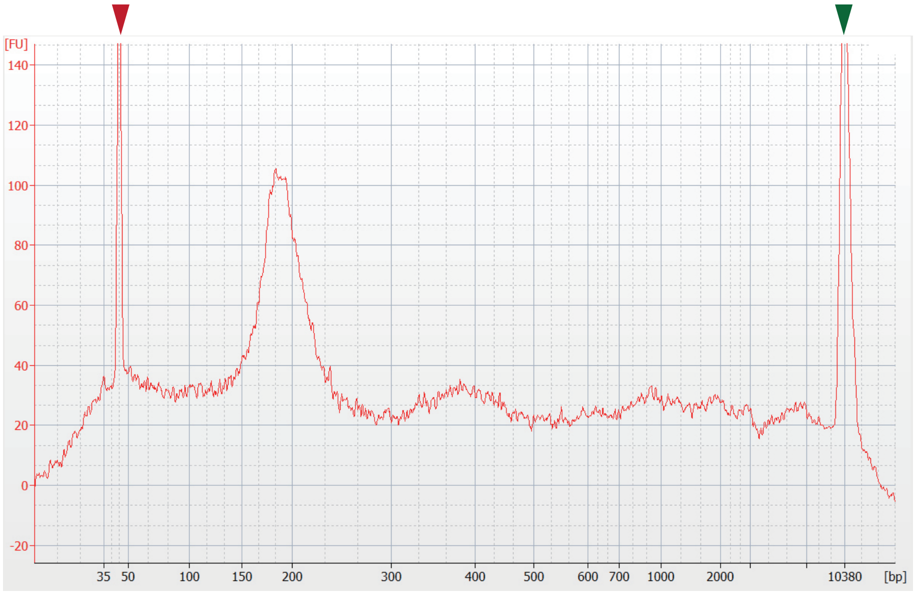

b

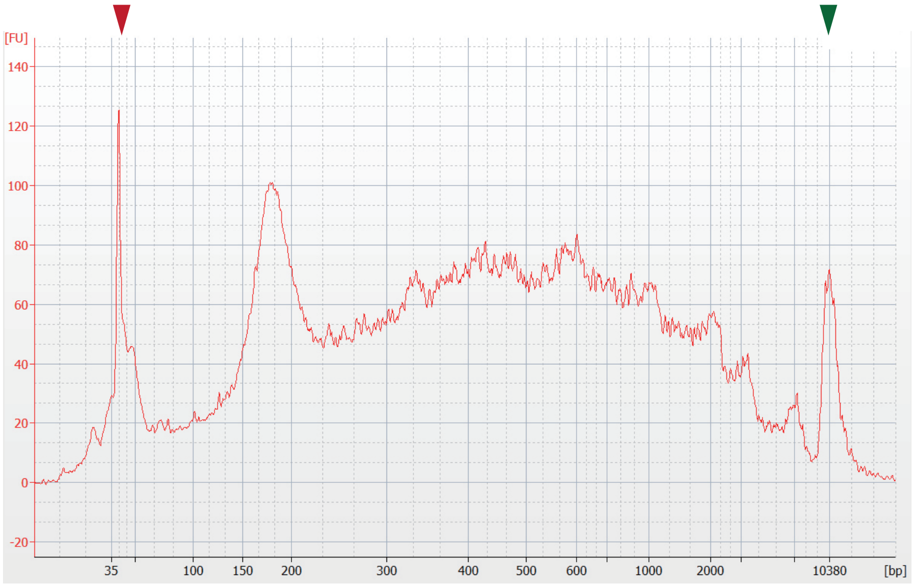

Supplementary Figure 3

Electrophoresis of the cell-free nucleic acids in FBS (**a**) and exosome-free FBS (**b**) was performed using a Bioanalyzer DNA HS tip. Cell-free nucleic acids with 1500 bp (green arrow) and 15 bp (red arrow) internal markers were resolved and quantified. X-axis represents the length of cell-free nucleic acids and internal markers. Y-axis represents fluorescence intensity. The concentration of cell-free nucleic acids in FBS and exosome-free FBS measured by Nanodrop were 30.08 ng/μL and 34.67 ng/μL, respectively.

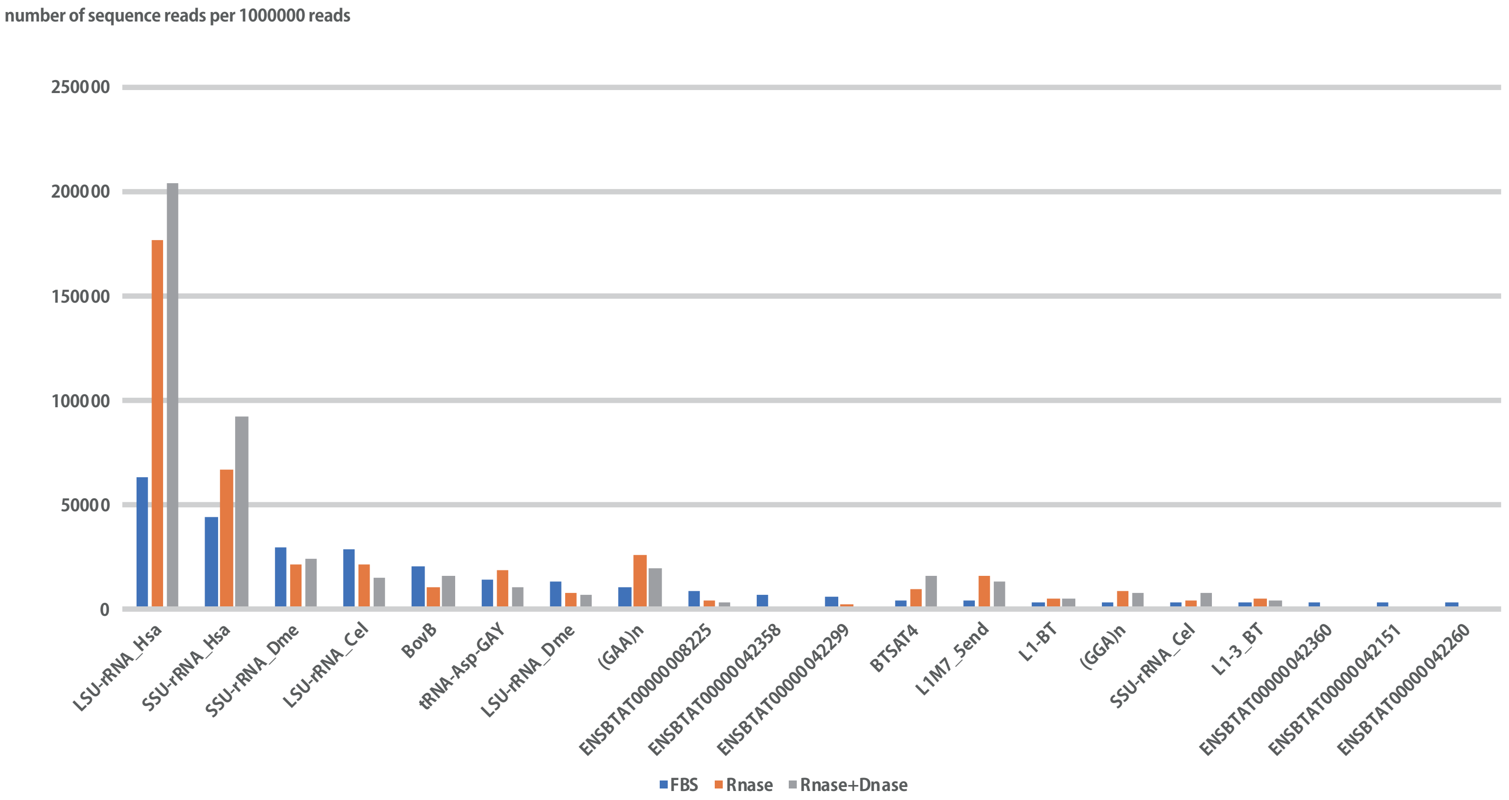

Supplementary Figure 4

Deep sequencing of exosomal RNAs in FBS

Exosomes were prepared from FBS by a stepwise centrifugation-ultracentrifugation method. After the collection of exosomes from FBS, exosomes were treated with RNase and DNase/RNase to remove extraneous nucleic acids outside of exosomes. Then, RNAs were extracted from the exosome fraction with or without RNase and RNase/DNase treatment, and RNA sequences were determined by next generation sequencing. Top 20 reads in exosomes without RNase and DNase treatment are presented on the X-axis. Retrotransposon RNAs, such as BovB and L1-BT, and the satellite RNA BTSAT4 were within the top 20 reads. These retrotransposon and satellite RNAs were comparable with those with RNase treatment and RNase/DNase treatment.

a

Peg10 WT

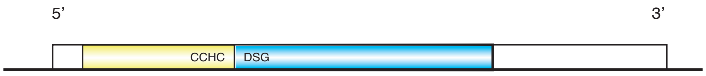


```
        10         20         30         40         50         60         70         80         90        100
MAAAGGSSNC PPPPPPPPPN NNNNNNTPKS PGVPDAEDDD ERRHDELPED INNFDEDMNR QFENMNLLDQ VELLAQSYSL LDHLDDFDDD DEDDDFDPEP

       110        120        130        140        150        160        170        180        190        200
DQDELPEYSD DDDLELQGAA AAPIPNFFSD DDCLEDLPEK FDGNPDMLGP FMYQCQLFME KSTRDFSVDR IRVCFVTSML IGRAARWATA KLQRCTYLMH

       210        220        230        240        250        260        270        280        290        300
NYTAFMMELK HVFEDPQRRE AAKRKIRRLR QGPGPVVDYS NAFQMIAQDL DWTEPALMDQ FQEGLNPDIR AELSRQEAPK TLAALITACI HIERRLARDA

       310        320        330        340        350        360        370
AAKPDPSPRA LVMPPNSQTD PTEPVGGARM RLSKEEKERR RKMNLCLYCG NGGHFADTCP AKASKNSPPG
```

b

Peg10ORF1-BTAUL1 fusion

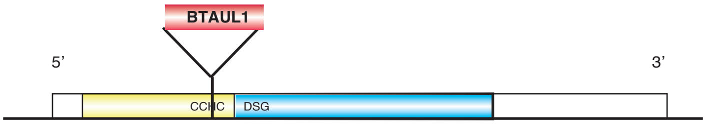


```
        10         20         30         40         50         60         70         80         90        100
MAAAGGSSNC PPPPPPPPPN NNNNNNTPKS PGVPDAEDDD ERRHDELPED INNFDEDMNR QFENMNLLDQ VELLAQSYSL LDHLDDFDDD DEDDDFDPEP

       110        120        130        140        150        160        170        180        190        200
DQDELPEYSD DDDLELQGAA AAPIPNFFSD DDCLEDLPEK FDGNPDMLGP FMYQCQLFME KSTRDFSVDR IRVCFVTSML IGRAARWATA KLQRCTYLMH

       210        220        230        240        250        260        270        280        290        300
NYTAFMMELK HVFEDPQRRE AAKRKIRRLR QGPGPVVDYS NAFQMIAQDL DWTEPALMDQ FQEGLNPDIR AELSRQEAPK TLAALITACI HIERRLARDA

       310        320        330        340        350        360        370        380        390
AAKPDPSPRA LVMPPNSQTD PTEPVGGARM RLSKEEKERR RKMNLCLYCA NAMWETWVQS LGWEDPLEKG KTTHSGILSW RIPWTV
```

Supplementary Figure 5

Schematic representation of the Peg10-BTAUL1 fusion protein generated from the capture of bovine retrotransposon at a DSB site. (**a**) Peg10 ORF1 protein sequence is presented in the yellow blue bar. (**b**) BTAUL1, a bovine SINE, was inserted into the Peg10 ORF1 protein and introduced 37 novel a.a. (red bar) in the Peg10 ORF1 protein sequence.
